# A High-Yielding Rice Cultivar “Takanari” Shows No N Constraints on CO_2_ Fertilization

**DOI:** 10.3389/fpls.2019.00361

**Published:** 2019-04-05

**Authors:** Toshihiro Hasegawa, Hidemitsu Sakai, Takeshi Tokida, Yasuhiro Usui, Hirofumi Nakamura, Hitomi Wakatsuki, Charles P. Chen, Hiroki Ikawa, Guoyou Zhang, Hiroshi Nakano, Miwa Yashima Matsushima, Kentaro Hayashi

**Affiliations:** ^1^Tohoku Agricultural Research Center, National Agriculture and Food Research Organization, Morioka, Japan; ^2^Institute for Agro-Environmental Sciences, National Agriculture and Food Research Organization, Tsukuba, Japan; ^3^Research Center for Agricultural Information Technology, National Agriculture and Food Research Organization, Tsukuba, Japan; ^4^Hokkaido Agricultural Research Center, National Agriculture and Food Research Organization, Memuro, Japan; ^5^Taiyo Keiki Co. Ltd., Toda, Japan; ^6^Department of Biology and Chemistry, Azusa Pacific University, Azusa, CA, United States; ^7^Kyushu Okinawa Agricultural Research Center, National Agriculture and Food Research Organization, Chikugo, Japan; ^8^Faculty of Horticulture, Chiba University, Matsudo, Japan

**Keywords:** chalky grains, climate change adaptation, CO_2_ fertilization effects, FACE, grain appearance quality, grain yield, nitrogen nutrition, *Oryza sativa*

## Abstract

Enhancing crop yield response to elevated CO_2_ concentrations (E-[CO_2_]) is an important adaptation measure to climate change. A high-yielding indica rice cultivar “Takanari” has recently been identified as a potential candidate for high productivity in E-[CO_2_] resulting from its large sink and source capacities. To fully utilize these traits, nitrogen should play a major role, but it is unknown how N levels influence the yield response of Takanari to E-[CO_2_]. We therefore compared grain yield and quality of Takanari with those of Koshihikari, a standard japonica cultivar, in response to Free-Air CO_2_ enrichment (FACE, +200 μmol mol^−1^) under three N levels (0, 8, and 12 g m^−2^) over three seasons. The biomass of both cultivars increased under E-[CO_2_] at all N levels; however, the harvest index decreased under E-[CO_2_] in the N-limited treatment for Koshihikari but not for Takanari. The decreased harvest index of Koshihikari resulted from limited enhancement of spikelet number under N-limitation. In contrast, spikelet number increased in E-[CO_2_] in Takanari even without N application, resulting in significant yield enhancement, averaging 18% over 3 years, whereas Koshihikari exhibited virtually no increase in yield in E-[CO_2_] under the N-limited condition. Grain appearance quality of Koshihikari was severely reduced by E-[CO_2_], most notably in N-limited and hot conditions, by a substantial increase in chalky grain, but chalky grain % did not increase in E-[CO_2_] even without N fertilizer. These results indicated that Takanari could retain its high yield advantage over Koshihikari with limited increase in chalkiness even under limited N conditions and that it could be a useful genetic resource for improving N use efficiency under E-[CO_2_].

## Introduction

Atmospheric CO_2_ concentrations ([CO_2_]) are rising at an unprecedented rate. Global [CO_2_] surpassed a milestone level of 400 ppm in 2015 and the rate of increase is becoming faster (https://www.esrl.noaa.gov/gmd/ccgg/trends/). Increasing concentrations of CO_2_ and other greenhouse gases will likely induce global environmental changes such as increases in temperatures, which are projected to have negative effects on food production of the major cereal crops (Zhao et al., 2017) or slow the rate of food production increase that is required to meet increasing demand (Iizumi et al., 2017). In contrast, increasing [CO_2_] will have a direct positive effect on crop photosynthesis and thereby increase grain yield; however, the size of the CO_2_ fertilization effect is one of the major sources of uncertainty in the projection of the global crop production (Rosenzweig et al., 2014; Deryng et al., 2016; Schleussner et al., 2018).

The uncertainty in projecting the CO_2_ fertilization effect arises partly from our limited quantitative understanding of crops to elevated [CO_2_] (E-[CO_2_]) (Hasegawa et al., 2017); however, CO_2_ fertilization effects themselves are variable in nature depending on various factors, including temperature, water, nutrition, and crop species or genotype (Kimball, 2016). To exploit the positive effects of E-[CO_2_] on crop production in the future, the mechanisms by which the CO_2_ fertilization effects vary must be better understood.

Application of nitrogen (N) fertilizers played a significant role in driving crop yields during the Twentieth century, together with genetic improvement for the suitability of a greater use of N (Evans, 1993; Cassman et al., 2003). Efficient use of N is required to reduce the negative impacts of agriculture on agricultural production not only for increasing farm economy but also for reducing environmental loads by agriculture via possible emission of N_2_O and contamination of groundwater (Zhang et al., 2015b).

Nitrogen is a key nutritional element mediating plant responses to E-[CO_2_]. Nitrogen often limits the enhancement of photosynthesis, biomass production, and yield by E-[CO_2_] (Nakano et al., 1997; Kimball et al., 2002). In fact, previous rice FACE experiments showed that grain yield enhancement in E-[CO_2_] decreased when N application was below 80 kg/ha (Hasegawa et al., 2017). Nutritional value and appearance quality of the grains are also vulnerable to change under E-[CO_2_] (Yang et al., 2007; Myers et al., 2014; Usui et al., 2016). Therefore, it is likely that N management practices will need to be revised or redesigned to achieve optimal productivity and quality outcomes under climate change (Bloom, 2015).

Genetic improvement is another major driver of improved crop productivity and will be the major and promising countermeasure against any negative effects of climate change. There is growing evidence of large variation in the yield response to E-[CO_2_] among cultivars. For instance, a rice FACE experiment in Japan revealed that a wide range of yield enhancement (3–36%) exists among cultivars and that higher-yielding cultivars with a large sink size had a greater [CO_2_] response (Hasegawa et al., 2013). A more recent study showed that quantitative trait loci for increasing spikelet number were effective in increasing grain yield under E-[CO_2_] (Nakano et al., 2017). Another rice FACE experiment in China also showed a large yield response to E-[CO_2_] with hybrid and inbred indica cultivars (Zhu et al., 2015).

These findings are encouraging in that improvement of yield potential can also be beneficial for positive yield response to E-[CO_2_]; however, yield improvement as a result of greater biomass generally requires greater nutrient input (Wang and Peng, 2017). We would therefore expect that the yield advantage would be smaller where the nutrient resource is limiting. However, previous cultivar studies of yield responses to E-[CO_2_] were conducted under conventional N conditions and the potential interactions among [CO_2_], cultivar, and N might have been overlooked.

E-[CO_2_] is known to reduce grain appearance quality (Yang et al., 2007), which is exacerbated by increases in temperature largely through the increased occurrence of chalky grains (CG) (Usui et al., 2016). The severity of quality degradation by E-[CO_2_] is cultivar dependent (Usui et al., 2014), and N dependent (Usui et al., 2016); however, the combined effects of N and [CO_2_] have not been tested to date.

In the present study, we focused on the following two cultivars with contrasting yield potential and appearance quality: Takanari, a high-yielding indica cultivar (Imbe et al., 2004) and Koshihikari, a standard japonica cultivar (Kobayashi et al., 2018). Takanari has several favorable traits under E-[CO_2_], including greater photosynthetic capacity (Chen et al., 2014), biomass, and grain yield (Hasegawa et al., 2013), and better appearance quality (Zhang et al., 2015a) with little increase in water use (Ikawa et al., 2018) compared to Koshihikari. Koshihikari has been a dominant rice cultivar in Japan for more than 50 years, accounting for more than a third of the rice planted area of the country (Kobayashi et al., 2018). Koshihikari has an intermediate response to E-[CO_2_] in key traits such as yield (Hasegawa et al., 2013), grain appearance quality (Usui et al., 2014) and grain nutritional quality (Myers et al., 2014) and so can serve as an appropriate check cultivar for the study. To design future N management for contrasting cultivars, field level responses to E-[CO_2_] must be compared. FACE is currently the most reliable technology to compare the [CO_2_] responsiveness of different cultivars in open fields. We therefore grew these cultivars under three different N levels for 3 years and compared yield and quality traits in response to E-[CO_2_] at the Tsukuba FACE facility in Japan.

## Materials and Methods

### Site Description and Weather Conditions

The experiments were conducted at the Tsukuba FACE facility, Tsukubamirai, Ibaraki, Japan (35°58′N, 139°60′E, 10 m above sea level) over 3 years (2012–2014). Details of the site are described by Nakamura et al. (2012) and Hasegawa et al. (2013). Briefly, the site was established in 2010 in the central region of Japan. The climate is humid subtropical with an average temperature of 13.8°C and annual precipitation of 1,280 mm. The soil is a Fluvisol, which is typical of alluvial areas with 23% clay and 40% silt contents. The total C and N concentrations were 21.4 and 1.97 mg g^−1^, respectively.

Average air temperatures over the three growing seasons were similar among the 3 years, ranging from 23.5 to 24.2°C (Table 1) and were higher than the 20 year (1991–2010) average of 23.5°C recorded at the nearby weather station, Tsukuba, located approximately 17 km away from the site (available http://www.data.jma.go.jp/obd/stats/etrn/). The solar radiation was between 17.1 and 18.8 MJ m^−2^d^−1^ (Table 1), which was 12% higher than the 20-year average of 15.9 MJ m^−2^d^−1^.

**Table 1:** Weather conditions and CO_2_ control during the three growing seasons at the Tsukuba FACE site.

**Year**	**Month**	**Air temperature (**^**°**^**C)**			**Solar radiation**	**Precipitation**	**CO**_**2**_ **concentration (μmol mol**^**−1**^**)**			
		**Mean**	**Max**	**Min**	**(MJ m^**−2**^ d^**−1**^)**	**(mm)**	**A-[CO**_**2**_**]**		**E-[CO**_**2**_**]**	
2012	May (23)^a^	19.2	23.8	15.3	21.4	26				
	June	19.8	23.4	16.7	18.1	219	383^b^	±9.5^c^	580	±13.7
	July	24.9	28.7	21.6	18.9	125	379	±10.6	571	±18.9
	August	26.8	31.3	22.8	20.0	22	383	±8.6	577	±11.6
	September (18)	25.7	30.7	21.7	16.4	60	396	±14.3	587	±16.6
	Growing season mean	23.8	28.0	20.2	18.8	451	383	±11.2	578	±15.7
2013	May (24)	20.1	24.2	16.4	20.1	11				
	June	21.3	24.9	18.4	16.8	99	389	±9.8	583	±11.4
	July	25.0	29.3	21.8	18.9	31	379	±10.9	572	±14.5
	August	26.9	31.8	23.0	19.5	106	381	±12.2	573	±20.0
	September(15)	25.1	29.2	21.8	14.2	139	386	±8.5	577	±10.1
	Growing season mean	24.2	28.4	20.8	18.0	387	384	±11.4	576	±15.5
2014	May (22)	20.7	25.7	16.3	21.8	35				
	June	22.0	25.4	19.2	16.8	282	384	±9.9	582	±11.3
	July	24.9	29.0	21.5	18.9	113	379	±11.1	579	±11.7
	August	25.7	29.4	22.4	16.0	147	375	±12.5	573	±25.0
	September(18)	21.7	25.6	18.4	13.6	77	384	±11.3	584	±16.7
	Growing season mean	23.5	27.4	20.3	17.1	653	380	±11.6	580	±17.4
	Three years growing season mean	23.9	27.9	20.4	18.0	497	382		578	

a *The numbers in the brackets for May and September are the day of month for transplant and harvest for Takanari*.

b *CO_2_ concentration in June includes a few days in May*.

c *Day-to-day standard deviation*.

### Treatments and Experimental Design

#### CO_2_ Treatment

We used four farmers' fields for the CO_2_ treatments, each of which had two octagonal plots that were of approximately 240 m^2^ (inner diameter of 17 m) in size; one for E-[CO_2_] and the other for ambient [CO_2_] (A-[CO_2_]) as the control plot, which were approximately 70 m apart to avoid contamination of the fumigated CO_2_ in the A-[CO_2_] plots. In the E-[CO_2_] plots, polyethylene emission tubes were installed horizontally on the peripheries of the octagonal plots. [CO_2_] was continuously measured at the center of the plots in both E-[CO_2_] and A-[CO_2_] plots with infrared gas analyzers (LI-820; LI-COR, Lincoln, NE, USA). We checked the quality of liquid CO_2_ supplied from the company (NIPPON EKITAN Corporation, Tokyo, Japan) every time it was delivered and confirmed that the concentration of ethylene was below the detectable level of 10 nmol mol^−1^. The emission tubes on each side were controlled independently with electric pneumatic regulators (ITV2031; SMC Corporation, Tokyo, Japan). Pure CO_2_ was fumigated in principle from three windward sides to keep [CO_2_] at the center of the plots, 200 μmol mol^−1^ above that in A-[CO_2_]. The accuracy and level of [CO_2_] were similar across the 3 years, and [CO_2_] in E-[CO_2_] was elevated by an average of 194, 192, and 195 μmol mol^−1^ in 2012, 2013, and 2014, respectively, which were slightly but consistently lower than the target elevation of 200 μmol mol^−1^ (Table 1).

#### Fertilizer Application, N Treatment, and Soil N Measurements

Nitrogen fertilizer treatment was similar to that reported by (Oikawa et al., 2017). Briefly, all plots received equal amounts of phosphorous (P) and potassium (K) as a PK compound fertilizer at 4.36 g m^−2^ of P and 8.30 g m^−2^ of K prior to flooding. After flooding and puddling (tillage practice with standing water), we placed 35 cm-wide PVC corrugate boarding horizontally to a depth of approximately 15 cm below the soil surface to separate three N fertilizer subplots in each CO_2_ main plot at 0, 8, and 12 g m^−2^ (hereafter 0, 8, and 12 N). In the 8 and 12 N plots, we then applied three forms of N as follows: urea and two types of coated urea 5 or 6 days prior to transplanting. The two types of coated urea were LP100 and LP140 (JCAM Agri. Co. Ltd., Tokyo, Japan) in 2012, and LP100 and LP40 (JCAM Agri. Co. Ltd.) in 2013 and 2014. The proportion of urea and LP100 in the total N applied was 25 and 50%, respectively. The remainder (25%) was applied as either LP140 (2012) or LP40 (2013 and 2014). No N fertilizer was applied to the 0N plot. The size of each treatment plot differed year-to-year, but was >15 m^2^ to accommodate at least two cultivars (see section Plant materials and cultivation methods). Each plot was puddled again for uniformity of fertilizer within each treatment.

In 2012, we collected soil samples from 0 to10 cm of the soil surface at transplanting. After passing the samples through a 2-mm sieve, we extracted exchangeable ammonium in a 1 M KCl solution. Ammonium N was determined in the filtered solution by the indophenol colorimetric method. We also conducted field soil incubation to estimate N supply in the soil in 2012. Before flooding in late April, we took soil samples from the plow layer of 8 N plots and passed them through a 2-mm sieve without air-drying. We then put the sieved unfertilized wet soil (10 g on the dry weight basis) into a 120-ml vial and added distilled water to make up the total amount of water including soil water to 20 ml. We sealed the vials after purging the headspace of the vial with N_2_ gas. The vials were then wrapped with aluminum foil to keep the contents dark and placed in the 8 N plot during the flooding period until late August. At harvest, we measured the exchangeable ammonium N. By subtracting the initial ammonium N at transplanting, we estimated N mineralized during the growing season.

### Plant Materials and Cultivation Methods

We used a high-yielding indica cultivar, Takanari, and a standard japonica cultivar, Koshihikari. Pregerminated seed of the two cultivars were sown in seedling trays (Minoru Pot 448, Minoru Industrial Co. Ltd. Okayama, Japan) filled with sterilized soil. The seedlings were raised in the puddled open field for approximately 4 weeks, with a tunnel cloche or floating mulch for the first 2 weeks. In each CO_2_ and N treatment plots, we manually transplanted these seedlings in late May at a spacing of 30 × 15 cm (22.2 hills m^−2^, 3 seedlings per hill). Dates of sowing and transplanting each year are provided in Table 2. The size of each cultivar in each N treatment varied but was >3 m^2^. The field was kept flooded until late August until the surface water was withdrawn for harvesting. The depth of the surface water was 5–10 cm. Herbicide, pesticide, and fungicide were applied as described previously (Hasegawa et al., 2013). In mid-July, just before the stem-elongation phase, we installed plant support netting made of polyethylene, with an aperture size of 30 cm, to prevent plants from lodging.

**Table 2:** The effects of two levels of [CO_2_] and three levels of nitrogen on heading date, maximum tiller number, and culm length ± standard deviation (n = 4) of Koshihikari and Takanari tested at Tsukuba FACE for three growing seasons.

**Cultivar**	**Year**	**Sowing date**	**Trans planting date**	**N (g m^**−2**^)**	**Days to heading**^**a**^		**Maximum tiller number (m**^**−2**^**)**		**Culm length (cm)**	
					**A-[CO_**2**_]**	**E-[CO_**2**_]**	**A-[CO_**2**_]**	**E-[CO_**2**_]**	**A-[CO_**2**_]**	**E-[CO_**2**_]**
Koshihikari	2012	24-Apr	23-May	0	71 ± 0.5	70 ± 1.0	377 ± 19	390 ± 38	77.8 ± 0.5	78.5 ± 1.3
				8	70 ± 0.0	70 ± 0.5	421 ± 61	556 ± 61	87.1 ± 3.1	89.1 ± 1.8
				12	71 ± 0.6	70 ± 0.5	601 ± 45	592 ± 69	96.4 ± 5.6	95.4 ± 3.7
	2013	23-Apr	23-May	0	72 ± 0.0	69 ± 1.8	357 ± 29	381 ± 16	83.1 ± 3.5	83.8 ± 1.7
				8	73 ± 0.6	72 ± 0.6	556 ± 22	584 ± 42	92.2 ± 1.9	97.2 ± 3.4
				12	ND^b^	ND	ND	ND	96.7 ± 5.1	99.3 ± 5.6
	2014	22-Apr	22-May	0	70 ± 1.0	66 ± 0.0	443 ± 40	404 ± 52	79.1 ± 1.7	76.7 ± 4.0
				8	71 ± 0.5	69 ± 1.0	671 ± 31	672 ± 26	92.2 ± 3.8	86.2 ± 1.1
				12	71 ± 1.0	68 ± 1.0	755 ± 49	771 ± 59	95.3 ± 1.9	89.8 ± 4.4
	Mean			0	71	68	392	392	80.0	79.6
				8	71	70	550	604	90.5	90.8
				12	71	69	678	681	96.1	94.8
Takanari	2012	24-Apr	23-May	0	ND	ND	ND	ND	63.0 ± 2.9	68.1 ± 3.1
				8	77 ± 0.6	77 ± 1.5	445 ± 50	457 ± 68	69.2 ± 3.9	68.7 ± 3.3
				12	ND	ND	ND	ND	ND	ND
	2013	23-Apr	23-May	0	77 ± 0.8	77 ± 1.0	290 ± 15	271 ± 15	64.2 ± 3.9	65.0 ± 1.6
				8	76 ± 0.5	76 ± 1.0	497 ± 28	456 ± 50	72.7 ± 1.6	75.7 ± 1.6
				12	ND	ND	ND	ND	80.6 ± 2.4	79.8 ± 3.9
	2014	22-Apr	22-May	0	76 ± 1.4	75 ± 1.5	304 ± 52	351 ± 40	60.5 ± 1.2	62.1 ± 1.2
				8	75 ± 1.5	73 ± 0.5	610 ± 32	676 ± 64	69.5 ± 0.9	71.0 ± 2.9
				12	74 ± 0.6	73 ± 0.5	629 ± 88	685 ± 123	71.3 ± 2.3	74.8 ± 2.9
	Mean			0	77	76	297	311	62.6	65.1
				8	76	75	518	530	70.5	71.8
				12	74	73	629	685	76.0	77.3
**ANOVA FOR THE 2014 DATA**^**c**^										
CO_2_					^***^		ns		ns	
N					ns		^***^		^***^	
N × CO_2_					ns		ns		ns	
Variety (V)					^***^		^***^		^***^	
V × CO_2_					^***^		^*^		^***^	
V × N					^***^		^*^		ns	
V × CO_2_ × N					ns		ns		ns	

a *Days from transplanting to heading*.

b *No data (not measured)*.

c *ANOVA was conducted for 2014 data only without missing cells*.

Statistically significant effects are indicated as

*** P < 0.001;

* *P < 0.05. ns, not significant*.

### Growth, Yield, and Quality Measurements

We measured the number of tillers of six hills in each treatment in each block every week from 2 weeks after transplanting until the heading stage and the maximum tiller number was determined as the largest number of tillers observed in these measurements. Culm length was measured for three hills in each plot by measuring the length from the shoot base to the panicle neck node. Panicle emergence was recorded every 2 to 3 days and the heading date was determined as the date when the number of emerged panicles reached 50% of the total productive stems in each treatment. We made all these measurements for 8 N in all years; however, we did not consistently collect all the data for 0 or 12N for all years, which are indicated as ‘ND' (i.e., no data) in Table 2.

At harvest, 18–21 hills from an area of 0.81–0.95 m^2^ in the middle of each plot were collected and air-dried under the rain shelter. We measured yield and yield components following the method described by Hasegawa et al. (2013). Briefly, we first measured air-dried aboveground biomass and moisture content for both grains and straws to estimate dry biomass at harvest. We counted the number of panicles and threshed spikelets to determine spikelet number and grain weight. A subsample of the unhulled spikelets were soaked in an ammonium sulfate solution with a specific gravity of 1.06 to determine the number of filled (sunken) or unfilled (floated) spikelets. Another subsample of spikelets were dehulled and the brown rice yield was determined. The yield was expressed on the 15% moisture content basis.

We also measured grain appearance quality using a subsample of 50 g of the unsieved brown rice with a grain quality inspector (RGH120A; Satake Corp., Hiroshima, Japan) equipped with image analysis software. The inspector classified each grain into such categories as undamaged, cracked, and chalked grains of different types depending on the position of chalk appearance on the grain. Because E-[CO_2_] is known to decrease the appearance quality of various cultivars via increased percentage of CG (Usui et al., 2014, 2016), we tested the effects of CO_2_, N, and varieties on the percentages of undamaged grains (UDG) and CG (sum of white belly, basal white, and milky white grains).

In addition to the 18–21 hills for yield determination, we collected 4–6 hills at the heading and maturity stages, which were separated by organs and oven-dried at 80°C to determine dry weights. The dried samples were ground and used for tissue N concentration measurements with an NC analyzer (Sumigraph NC-22F, Sumika Chemical Analysis Service, Tokyo, Japan). We did not measure panicle N concentration (PNC) of Takanari in 12N in 2012 and 2013 or appearance quality in 2012.

### Experimental Design and Statistical Analysis

The experimental plots were laid out in a split-split plot design with CO_2_ as the main plot, N as the split plot, and cultivar as the split-split plot with four replicates. Analysis of variance (ANOVA) for the year, CO_2_, N, and cultivar effects was conducted with the statistical package R, version 3.3.3, using a split-split-split procedure described by de Mendiburu (2017). For the traits with missing cells, we performed ANOVA only for the years when full datasets were available.

## Results

### Soil N, Growth, and Development

Soil exchangeable ammonium N (EAN) at transplanting in the 0N plot in 2012 was 1.53± 0.32 and 1.59 ± 0.28 g m^−2^ in A-[CO_2_] and E-[CO_2_], respectively. Soil N mineralized during the flooding period in 2012 was 5–6 times greater than the initial EAN and not significantly different between the CO_2_ treatments: 8.25± 0.73 g m^−2^ in A-[CO_2_] and 10.0 ± 1.45 g m^−2^ in E-[CO_2_].

Days to heading (DTH) were on average approximately 5 days longer for Takanari than for Koshihikari (*P* < 0.001, Variety main effect, Table 2). Elevated [CO_2_] decreased DTH significantly by 1–3 days (*P* < 0.001), which was similar to previous observations in other rice FACE experiments (Hasegawa et al., 2016); however, the effect differed between the two varieties with a significant interaction between CO_2_ and variety (*P* < 0.001). DTH was decreased by as much as 4 days in Koshihikari but only by 1–2 days in Takanari. Maximum tiller number (MTN) also differed significantly between the two cultivars with Koshihikari having approximately 10% greater MTN than did Takanari (*P* < 0.001, Table 2). The effect of E-[CO_2_] on MTN was not apparent in Koshihikari but was positive in Takanari, which resulted in a significant Variety × CO_2_ interaction (*P* < 0.05).

Culm length at harvest was much longer in Koshihikari than in Takanari by as much as 20 cm (*P* < 0.001). The ANOVA result for culm length only showed a strong interaction between CO_2_ and cultivar for the 2014 data, where culm length of Koshihikari was decreased by E-[CO_2_] but that of Takanari was increased (Variety × CO_2_, *P* < 0.001, Table 2). This strong interaction was not observed in other years. The effect of N was significantly positive for MTN and culm length. DTH and MTN showed a significant variety × N interaction.

### Yield and Yield Components

Elevated [CO_2_] increased paddy (unhulled grain) yield by 78 g m^−2^ (9.3%) and aboveground dry mass by 139 g m^−2^ (10.0%) when averaged over years, N, and cultivars (*P* < 0.001, Table 3). In all treatments, Takanari had a significantly greater paddy yield than did Koshihikari (*P* < 0.001, Variety main effect) and the difference was greater in E-[CO_2_] (*P* < 0.001, Variety × CO_2_). The greatest yield enhancement of paddy yield by E-[CO_2_] was observed in Takanari in the 0 N plot (18.0% averaged over 3 years, Table 3), and the smallest enhancement was in Koshihikari in the same 0N plot, resulting in a significant CO_2_ × N × Variety interaction (0% averaged over 3 years, *P* = 0.080). The enhancement of aboveground dry mass by E-[CO_2_] was higher in Takanari than in Koshihikari (*P* < 0.05, CO_2_ × Variety) similarly across N treatments with no significant three-way interaction. The harvest index (HI) of Takanari was much greater than that of Koshihikari by as much as 9 points on average and was not affected by [CO_2_] or N applications (Table 3). In contrast, the HI of Koshihikari was decreased by E-[CO_2_] particularly in the 0N plot by 3 points. This resulted in a significant three-way interaction effect (CO_2_ × N × Variety) on HI (*P* < 0.05).

**Table 3:** The effects of two levels of [CO_2_] and three levels of nitrogen on paddy yield, aboveground biomass, and harvest index of Koshihikari and Takanari tested at Tsukuba FACE for three growing seasons.

**Cultivar**	**Year**	**N (g m^**−2**^)**	**Paddy Yield**^**a**^**(g m**^**−2**^**)**		**Aboveground dry mass (g m**^**−2**^**)**		**Harvest index**^**b**^**(%)**	
			**A-[CO_**2**_]**	**E-[CO_**2**_]**	**A-[CO_**2**_]**	**E-[CO_**2**_]**	**A-[CO_**2**_]**	**E-[CO_**2**_]**
Koshihikari	2012	0	476	477	925	954	44	42
		8	756	851	1,323	1,471	49	49
		12	882	950	1,437	1,627	52	50
	2013	0	583	592	1,104	1,206	45	42
		8	817	885	1,532	1,660	45	45
		12	869	948	1,594	1,770	46	46
	2014	0	571	515	1,004	978	48	45
		8	817	836	1,448	1,484	48	48
		12	845	954	1,524	1,661	47	49
	Mean	0	543	528	1,011	1,046	46	43
		8	797	857	1,434	1,538	47	47
		12	865	950	1,518	1,686	49	48
Takanari	2012	0	608	691	912	1,035	57	57
		8	1065	1,132	1,541	1,676	59	57
		12	1156	1,263	1,797	2,022	55	53
	2013	0	698	780	1,076	1,231	55	54
		8	1061	1,183	1,621	1,844	56	55
		12	1058	1,245	1,734	1,953	52	54
	2014	0	623	802	951	1,198	56	57
		8	1102	1,167	1,644	1,735	57	57
		12	1192	1,311	1,816	1,979	56	56
	Mean	0	643	758	979	1,155	56	56
		8	1076	1,160	1,602	1,752	57	56
		12	1135	1,273	1,782	1,985	54	55
**ANOVA**								
Year			^*^		^**^		^***^	
CO_2_			^***^		^***^		ns	
CO_2_ × Year			ns		ns		ns	
N			^***^		^***^		^***^	
N × CO_2_			ns		ns		ns	
N × Year			^*^		^*^		^*^	
N × Year × CO_2_			ns		ns		0.067	
Variety (V)			^***^		^***^		^***^	
V × Year			0.079		^**^		ns	
V × CO_2_			^***^		^*^		0.068	
V × N			^***^		^***^		^***^	
V × Year × CO_2_			ns		ns		ns	
V × Year × N			ns		ns		^***^	
V × CO_2_ × N			0.080		ns		^*^	
V × CO_2_ × N × Year			ns		ns		ns	

a *Expressed on the 15% moisture content*.

b *Paddy yield divided by aboveground biomass both on the 0% moisture*.

Statistically significant effects are indicated as

*** P < 0.001;

** P < 0.01;

* *P < 0.05. The value indicates the probability between 0.05 and 0.1. ns, not significant*.

The response of hulled grain (brown rice) yield to CO_2_ and N was similar to that of paddy yield (Table 4). Takanari had a greater brown rice yield in E-[CO_2_] than Koshihikari and the enhancement by E-[CO_2_] was particularly high in the 0N plot (CO_2_ × Variety × N, *P* = 0.057). Elevated [CO_2_] increased sink-related yield components such as panicle density, spikelet per panicle, and spikelet density (CO_2_ main effect, *P* = 0.098, < 0.05 and < 0.01, respectively); however, the effects of E-[CO_2_] on spikelet per panicle and spikelet density was different between cultivars (CO_2_ × Variety, *P* < 0.05). These yield components were increased by E-[CO_2_] by 9 and 11%, respectively, in Takanari; however, virtually no increase was found in Koshihikari. High N application did not moderate the response of these yield components to E-[CO_2_]. In contrast, a significant three-way interaction was observed in the percentage of ripened spikelets (*P* < 0.05).

**Table 4:** The effects of two levels of [CO_2_] and three levels of nitrogen treatment on yield and yield components of Koshihikari and Takanari tested at Tsukuba FACE for three growing seasons.

**Cultivar**	**Year**	**N (g m^**−2**^)**	**Brown rice yield**^**a**^**(g m**^**−2**^**)**		**Panicle density (m**^**−2**^**)**		**Spikelet per panicle**		**Spikelet density (10**^**3**^**m**^**−2**^**)**		**Percentage of ripened spikelet**		**Single grain mass (mg)**	
			**A-[CO_**2**_]**	**E-[CO_**2**_]**	**A-[CO_**2**_]**	**E-[CO_**2**_]**	**A-[CO_**2**_]**	**E-[CO_**2**_]**	**A-[CO_**2**_]**	**E-[CO_**2**_]**	**A-[CO_**2**_]**	**E-[CO_**2**_]**	**A-[CO_**2**_]**	**E-[CO_**2**_]**
Koshihikari	2012	0	375	372	256	263	77	77	19.8	20.1	86.5	84.2	22.1	22.0
		8	604	679	379	407	82	86	31.1	35.1	87.2	86.5	22.1	21.7
		12	709	762	431	440	88	88	38.1	38.9	82.1	86.4	21.7	22.2
	2013	0	453	453	290	305	84	83	24.5	25.3	85.0	77.9	19.8	19.6
		8	646	697	387	415	88	91	33.9	37.7	83.7	80.6	20.4	19.9
		12	686	746	409	423	91	95	37.1	40.3	78.6	81.7	20.4	20.0
	2014	0	462	405	278	252	84	86	23.4	21.6	85.9	80.5	21.4	20.9
		8	656	673	396	368	90	95	35.7	35.0	76.7	81.4	21.4	21.3
		12	678	769	451	456	91	94	41.2	42.9	66.8	73.5	21.1	20.8
Mean		0	430	410	275	273	82	82	22.5	22.4	85.8	80.8	21.1	20.8
		8	635	683	387	396	87	91	33.6	35.9	82.6	82.8	21.3	21.0
		12	691	759	430	440	90	93	38.8	40.7	75.8	80.6	21.1	21.0
Takanari	2012	0	448	511	211	222	131	135	27.4	30.2	84.4	87.1	19.1	19.8
		8	807	853	285	289	159	172	45.4	49.6	86.3	85.2	19.8	19.9
		12	889	967	260	269	195	205	50.7	54.9	82.4	81.6	20.4	20.7
	2013	0	514	572	228	239	134	147	30.6	35.0	83.9	80.9	19.2	19.1
		8	804	900	272	285	163	175	44.5	49.8	82.6	81.6	20.4	20.3
		12	784	949	292	304	166	179	48.3	54.6	69.6	79.0	20.5	20.5
	2014	0	476	616	196	222	143	158	28.1	35.1	84.2	84.5	19.0	19.5
		8	848	896	259	270	189	193	49.1	52.2	74.5	75.8	20.3	20.2
		12	917	1013	271	291	199	202	53.9	58.7	71.6	74.1	20.6	20.8
mean		0	479	566	211	228	136	147	28.7	33.4	84.2	84.2	19.1	19.4
		8	820	883	272	281	171	180	46.3	50.5	81.1	80.9	20.2	20.1
		12	863	977	274	288	187	196	51.0	56.0	74.5	78.2	20.5	20.6
**ANOVA**														
Year			^*^		ns		^***^		^**^		^***^		^***^	
CO_2_			^***^		0.098		^*^		^**^		ns		ns	
CO_2_ × Year			ns		ns		ns		ns		ns		0.087	
N			^***^		^***^		^***^		^***^		^***^		^***^	
N × CO_2_			0.079		ns		ns		ns		^***^		ns	
N × Year			^*^		^**^		^**^		^**^		^***^		^***^	
N × Year × CO_2_			ns		ns		ns		ns		^*^		ns	
Variety (V)			^***^		^***^		^***^		^***^		0.055		^***^	
V × Year			0.056		ns		^***^		ns		ns		^***^	
V × CO_2_			^***^		ns		^*^		^**^		ns		^**^	
V × N			^***^		^***^		^***^		^***^		^*^		^***^	
V × Year × CO_2_			ns		^**^		ns		ns		0.066		ns	
V × Year × N			ns		^***^		^**^		ns		^***^		ns	
V × CO_2_ × N			0.057		ns		ns		ns		^*^		ns	
V × CO_2_ × N × Year			ns		ns		ns		ns		ns		ns	

a *Unsieved brown rice yield expressed on the 15% moisture content*.

Statistically significant effects are indicated as

*** P < 0.001;

** P < 0.01;

* *P < 0.05. The value indicates the probability between 0.05 and 0.1. ns, not significant*.

### Relationship Between N Uptake and Yield Components

**Figure 1:**
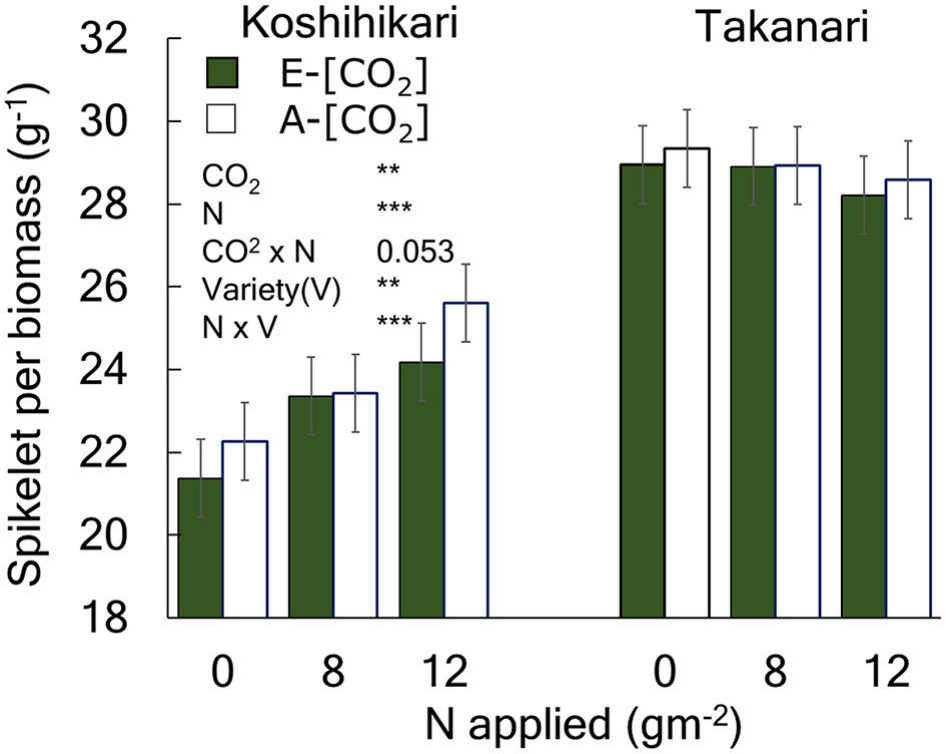
The effects of N and [CO_2_] on the spikelet number per unit aboveground biomass of Koshihikari and Takanari. The 3-year averages and a summary of ANOVA results with significant treatment and variety effects are shown in this figure. All data and the ANOVA results are provided in Table S2. ^***^ and ^**^ indicate significant differences at *P* = 0.001 and 0.01, respectively. The value indicates the probability between 0.05 and 0.1. Vertical bars are the standard errors of the residual of the ANOVA.

**Figure 2:**
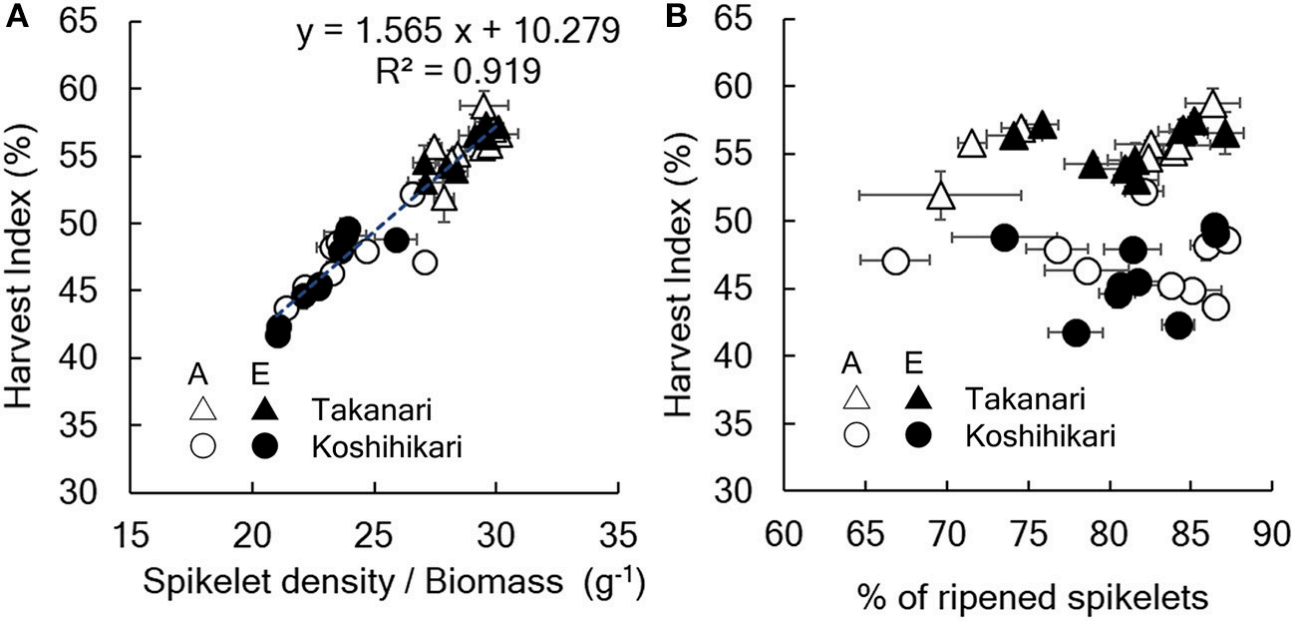
Relationship between harvest index and **(A)** spikelet number/aboveground biomass or **(B)** % of ripened spikelets. A, A-[CO_2_] = ambient [CO_2_]; E, E-[CO_2_] = elevated [CO_2_]. Vertical or horizontal bars are the standard errors of the mean in each year.

The yield enhancement of Koshihikari by E-[CO_2_] was substantially decreased by the limited N supply because HI decreased with a decrease in N supply significantly in E-[CO_2_]. In contrast, the HI of Takanari remained unchanged across the different N and CO_2_ treatments, which resulted in a significant difference in the CO_2_ × N response between the two cultivars. To explore the reason for this difference, we examined the relationship between HI and the sink-related traits. Spikelet number per unit aboveground biomass of Koshihikari was significantly smaller than that of Takanari (Figure 1, Table S1, Variety main effect, *P* < 0.01) and decreased under the reduced supply of N, particularly in E-[CO_2_]; however, that of Takanari was unchanged by the N conditions, resulting in a significant Variety × N interaction (*P* < 0.001). The HI was significantly correlated with the spikelet number per biomass (Figure 2A, *P* < 0.001); however, it was not correlated with the percentage of ripened spikelets (Figure 2B).

**Figure 3:**
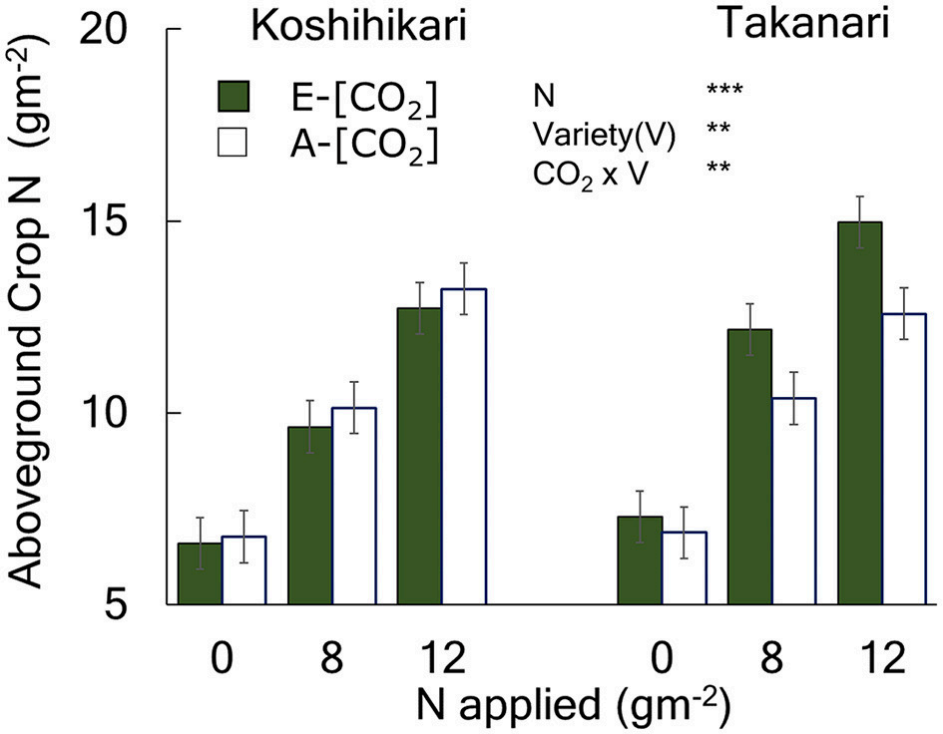
The effects of N and [CO_2_] on the aboveground crop N of Koshihikari and Takanari measured at maturity. The 2014 data with full data sets without missing cells are presented with a summary of ANOVA results with significant effects. All available data are presented in Table S2 along with a summary of ANOVA using 0 and 8N over the 3-year period which agreed with that in 2014 without significant interactions with year. *** and ** indicate significant differences at *P* = 0.001 and 0.01, respectively. Vertical bars are the standard errors of the residual of the ANOVA.

Aboveground crop N (ACN) increased with N supply (Figure 3, Table S1, N main effect, *P* < 0.001). Takanari had a greater ACN than Koshihikari at all N levels (Variety main effect, *P* < 0.01). The effect of E-[CO_2_] on ACN differed between cultivars; E-[CO_2_] did not affect ACN of Koshihikari but increased that of Takanari at all N levels, as indicated by a significant Variety × CO_2_ interaction (*P* < 0.01).

**Figure 4:**
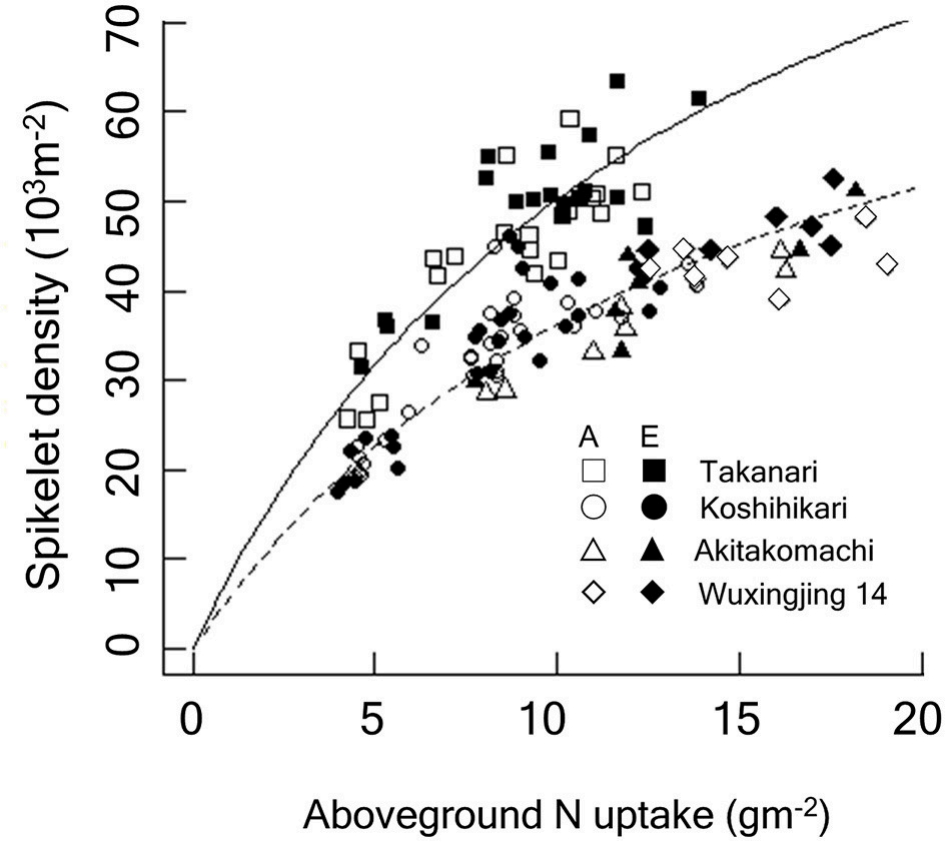
Relationship between spikelet density and crop N accumulated by the heading stage of Koshihikari and Takanari grown at the Tsukuba FACE for 3 years (2012–2014). Additionally, data from the Shizukuishi FACE, Iwate, Japan (“Akitakomachi”) and from the Wuxi FACE, Jiangsu, China (“Wuxianjing 14”) are shown (Kim et al., 2003; Yang et al., 2006). A linearized form of the Michaelis-Menten type rectangular hyperbola function was fitted to the data by the ordinary least square method. The regression lines were significantly different between Koshihikari and Takanari (*P* < 0.001) and between Takanari and the rest (*P* < 0.001). A, A-[CO_2_]; E, E-[CO_2_]. In the figure, regressions reconverted to the original form are shown: Solid, Takanari, y = 120.5x/(13.98 + x), *R*^2^ = 0.804, *P* < 0.001; Dashed, the rest, y = 90.11x/(14.89 + x), *R*^2^ = 0.876, *P* < 0.001.

We observed a close relationship between spikelet density and ACN at heading and harvest; however, the relationship was clearly cultivar-dependent (Figure 4), with Takanari having a substantially greater spikelet production capacity per unit N uptake than Koshihikari. For each cultivar, the spikelet density-crop N relationship was consistent across the different [CO_2_] treatments. In contrast, spikelet density of Takanari increased in response to E-[CO_2_] by increasing ACN even under N-limited conditions.

### Panicle N Concentration and Grain Appearance Quality

Panicle N concentration (PNC) increased with N supply (*P* < 0.001, Table 5); however, PNC was generally higher in Koshihikari than in Takanari (*P* < 0.001). For PNC, the ANOVA was based on the 2014 data only because of missing observations in the other 2 years. E-[CO_2_] decreased the PNC in most treatments; however, this decrease was larger in Koshihikari than that in Takanari (Variety × CO_2_, *P* < 0.01). Elevated [CO_2_] reduced the percentage of UDG severely, mainly owing to the increased CG (*P* < 0.001, Table 5). The two cultivars also showed a contrasting response to E-[CO_2_] for UDG and CG (CO_2_ × Variety, *P* < 0.001, Table 5). The decrease in UDG by E-[CO_2_] averaged 13.9 points for Koshihikari and 3.1 points for Takanari (Table 5), although UDG of Takanari was generally poor because of the high percentage of malformed grains across different CO_2_, N and years (data not shown). The decreases in UDG were mostly accounted for by the increases in CG (96%), which was mainly caused by the occurrence of white-base type CG (71% for Koshihikari and 33% for Takanari, Table S2). Nitrogen application significantly increased UDG and decreased CG (*P* < 0.001); however, the dependence on N application was larger in Koshihikari than in Takanari (Variety × N, *P* < 0.001, Table 5). Koshihikari had the poorest appearance quality in the 0 N plot, whereas Takanari showed the best appearance in the 0 N plot.

**Table 5:** The effects of two levels of [CO_2_] and three levels of nitrogen treatment on panicle N concentration, % of undamaged grains, and chalky grains of Koshihikari and Takanari tested at Tsukuba FACE for three growing seasons.

**Cultivar**	**Year**	**N (g m^**−2**^)**	**Panicle N concentration**		**Undamaged grain**		**Chalky grain**		**Heatdose (>26)**^**a**^	
			**(mg g**^**−1**^**)**		**(%)**		**(%)**		(^**°**^**C d**)	
			**A-[CO_**2**_]**	**E-[CO_**2**_]**	**A-[CO_**2**_]**	**E-[CO_**2**_]**	**A-[CO_**2**_]**	**E-[CO_**2**_]**	**A-[CO_**2**_]**	**E-[CO_**2**_]**
Koshihikari	2012	0	8.6	8.4	70.7	58.3	14.1	24.2	16.9	16.8
		8	9.6	9.0	74.7	68.4	8.7	14.6	16.9	16.8
		12	10.7	9.4	70.2	68.9	12.7	15.1	16.9	16.8
	2013	0	8.6	8.1	25.4	12.5	51.5	59.9	39.9	38.1
		8	9.6	8.9	46.1	29.4	33.3	50.1	39.9	39.7
		12	ND^b^	ND	45.2	26.1	33.8	55.4	(39.9)	(39.7)
	2014	0	8.3	8.1	59.5	34.1	28.4	52.2	25.9	23.8
		8	9.4	8.6	60.8	44.7	21.4	37.8	26.7	24.2
		12	10.3	9.1	61.0	46.6	17.6	35.4	26.7	24.2
Takanari	2012	0	8.7	8.4	55.9	52.5	4.6	4.8	(19.4)	(19.4)
		8	9.0	8.6	56.9	59.0	7.7	8.3	19.4	19.4
		12	ND	ND	ND	ND	ND	ND	(19.4)	(19.4)
	2013	0	8.6	8.2	45.1	44.0	13.1	15.6	35.5	37.2
		8	9.3	8.9	49.1	42.2	16.4	21.0	38.4	38.4
		12	ND	ND	46.4	43.4	12.3	19.9	(38.4)	(38.4)
	2014	0	8.7	8.0	48.5	42.5	8.8	17.4	14.9	21.9
		8	9.1	8.8	45.1	43.3	17.0	19.7	21.9	24.7
		12	9.3	9.2	47.0	42.9	16.7	23.9	24.7	24.7
ANOVA^c^			2014 data		2013–2014 data		2013–2014 data			
Year			^**^		^**^					
CO_2_			^*^		^***^		^***^			
CO_2_ × Year			ns		ns					
N			^***^		^***^		^***^			
N × CO_2_			ns		ns		ns			
N × Year			^**^		ns					
N × Year × CO_2_			^*^		^**^					
Variety (V)			ns		^***^		^***^			
V × Year			^***^		^***^					
V × CO_2_			0.098		^***^		^***^			
V × N			0.064		^***^		^***^			
V × Year × CO_2_			ns		ns					
V × Year × N			^*^		^*^					
V × CO_2_ × n			^*^		ns		ns			

a *Cumulative temperature above 26°C for 20 days after heading. The values in the brackets are estimated heatdose assuming that heading dates are the same as those in 8N of the same [CO_2_] and cultivar*.

b *No data (not measured)*.

c *ANOVA was conducted for the years without missing cells*.

Statistically significant effects are indicated as

*** P < 0.001;

** P < 0.01;

* *P < 0.05. The value indicates the probability between 0.05 and 0.1. ns, not significant*.

There was a significant year effect on both UDG and CG with the 2013 crop showing the poorest appearance quality (lowest UDG and highest CG) among the 3 years (*P* < 0.001, Table 5). The large year-to-year variations in UDG and CG were closely associated with the cumulative temperature above 26°C for 20 days after heading (early grain filling period), which was highest around 40°C d in 2013 and lowest around 17–19°C d in 2012 (Table 5). The effect of N on UDG and CG was largest in 2013 when the quality loss was largest in A-[CO_2_]. However, in E-[CO_2_], CG exceeded 50% in all three N treatments in Koshihikari. The differences in UDG and CG between the two cultivars were noted in 2013 (Variety × Year × N, *P* < 0.05), where CG of Koshihikari was larger than that of Takanari by as much as 40 points in the 0 N plot.

**Figure 5:**
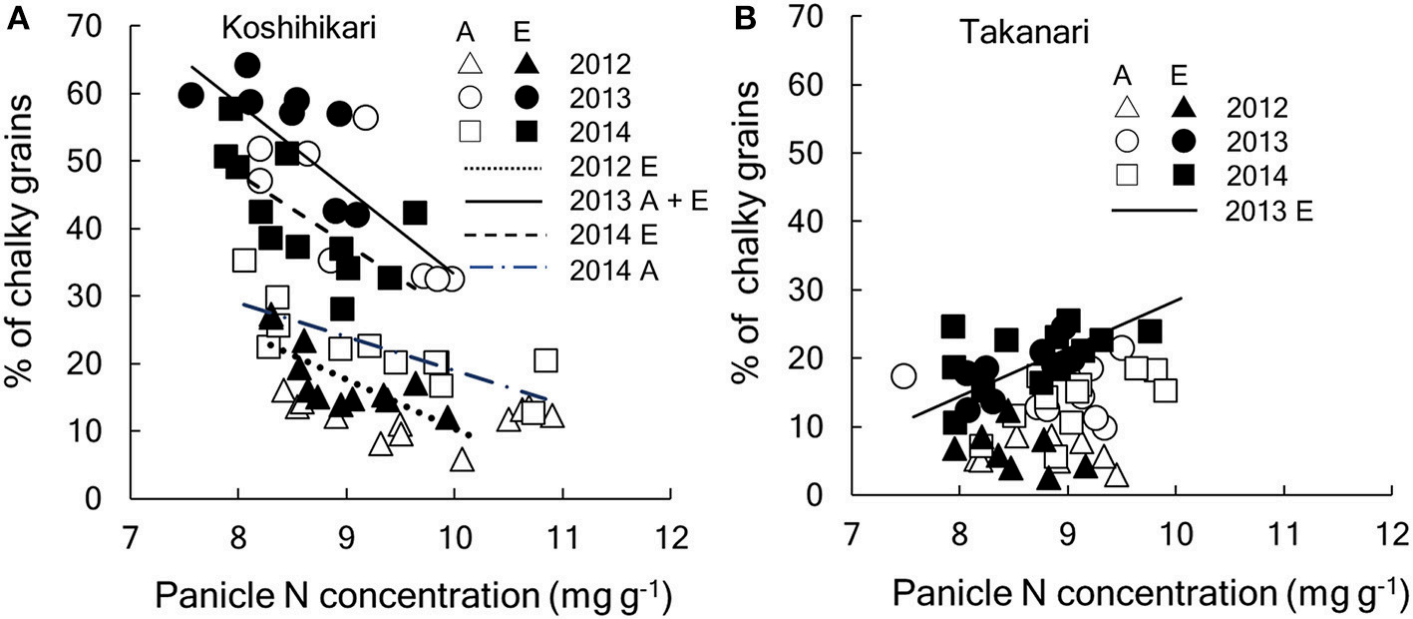
Relationship between percentage of chalky grains and panicle N concentration of **(A)** Koshihikari and **(B)** Takanari cultivars. Regressions lines were tested between A-[CO_2_] and E-[CO_2_] each year. In the figure, regression that are significant are drawn. If the [CO_2_] effect is significant, separate lines are drawn for each [CO_2_] and, if not, a common line is drawn. **(A)** Koshihikari, dotted, y = −7.32x + 83.5 for E-[CO_2_] in 2012 (*R*^2^ = 0.519, *P* < 0.01); solid, y = −12.6x + 159.3 for A- and E-[CO_2_] in 2013 (*R*^2^ = 0.580, *P* < 0.001);dot-dash y = −5.0x + 69.1 for A-[CO_2_] in 2014 (*R*^2^ = 0.613, *P* < 0.01); dashed, y = 10.3x + 130.3 for E-[CO_2_] in 2014 (*R*^2^ = 0.419, *P* < 0.05): **(B)** Takanari, y = 7.0x−41.4 (*R*^2^ = 0.474, *P* < 0.05). A, A-[CO_2_]; E, E-[CO_2_].

These significant interactions for the grain quality related traits are summarized in Figure 5. CG of Koshihikari varied significantly among years but significant negative relationships were observed within each year (Figure 5A). The regression lines were statistically different between A-[CO_2_] and E-[CO_2_] except for in 2013, suggesting that the reduction in PNC was not enough to fully explain the variation in CG due to different [CO_2_]. In contrast, CG of Takanari varied little among years and no negative relationship was observed with PNC, with a slightly positive relationship observed on one occasion (E-[CO_2_] in 2014, Figure 5B).

## Discussion

### CO_2_ Fertilization Effects Influenced by N and Cultivars

The limitation of CO_2_ fertilization effects as a result of limited N availability has long been known at the leaf physiological level or crop production level (Stitt and Krapp, 1999; Kimball et al., 2002). The present study has shown that the N-limited CO_2_ fertilization effect does not hold for Takanari, a high-yielding indica cultivar. Our 3-year FACE experiments with three N levels confirmed that yield enhancement of Koshihikari, a standard japonica rice cultivar, by E-[CO_2_] was limited by the soil N availability. The 3-year average yield enhancement of Koshihikari was 0%, whereas that in the standard and high levels of N was 10% (Table 3). We also confirmed that the N limited CO_2_ fertilization effect on grain yield was mainly via reduced biomass allocation to the grains (i.e., HI) rather than limited biomass increase at E-[CO_2_] as was found in previous rice FACE experiments (Kim et al., 2003; Kobayashi et al., 2006; Zhang et al., 2013). Ample evidence exists that the biomass response to E-[CO_2_] is limited by soil N availability in various plant species (Reich and Hobbie, 2012; Feng et al., 2015), which are in agreement with the physiological observations that the enhancement of leaf photosynthesis or canopy light use efficiency was decreased owing to poor N status of the leaves (Nakano et al., 1997; Sakai et al., 2006). In the present study, a relatively large amount of N was taken up by the crops even when no N fertilizer was applied, averaging 6.6 g m^−2^ for 100–110 days. This is similar to the typical rice paddy soil fertility in Japan (Toriyama, 2002) and the present N limitation might not have been so severe as to limit biomass enhancement in the E-[CO_2_] treatment.

The HI of Koshihikari was decreased by E-[CO_2_] under N-limited conditions because of the sink limitation. Elevated [CO_2_] increased biomass even in the N-limited condition; however, it did not increase spikelet number, and thereby reduced the sink: biomass ratio (Figures 1, 2A). This sink limitation did not occur in Takanari even without N application. Consequently, Takanari had a significant yield increase of 18% by E-[CO_2_] in the 0 N plot averaged over 3 years, whereas Koshihikari had virtually no increase in yield.

The major determinant of spikelet density is crop N uptake (Wada, 1969). We also confirmed the close positive association between crop N at the heading stage and spikelet number. In Figure 4, we included data from previous FACE experiments conducted in Shizukuishi, Japan and in Wuxi, China with 3 N levels over 3 years (Kim et al., 2003; Yang et al., 2006), both of which used japonica cultivars: Akitakomachi in Shizukuishi and Wuxianjing 14 in Wuxi. Spikelet density of the three japonica-type cultivars fell on the same response function to crop N uptake regardless of the [CO_2_] or N treatments, suggesting a strong limitation of spikelet production by crop N availability. The spikelet density of Takanari was also dependent on N but was consistently greater than that of Koshihikari at the same crop N for the entire range of crop N. Takanari is known for its high N use efficiency (Taylaran et al., 2009), and this characteristics was maintained in E-[CO_2_].

The large spikelet density of Takanari apparently resulted from a larger number of spikelets per panicle rather than from panicle density (Table 4). A previous study revealed that Takanari possess at least two genes that increase spikelet number per panicle: *APO1* and *GN1a* (Takai et al., 2014, 2018). These genes have proven to be effective under E-[CO_2_] (Nakano et al., 2017). The present study showed that the advantage was retained under the limited N condition.

The greater yield or spikelet response of Takanari to E-[CO_2_] compared to Koshihikari resulted from the enhanced crop N uptake even without N fertilizer application (Figure 3). Initially, we hypothesized that the advantage of high-yielding Takanari in response to E-[CO_2_] over Koshihikari was pronounced with greater N supply because a limited availability of N would not meet the high N demand by Takanari. However, this hypothesis was not supported by our results; crop N uptake of Takanari was enhanced by E-[CO_2_] even in the 0N plot, which in turn increased spikelet density (Figure 4), whereas that of Koshihikari was not. The source of additional N uptake by E-[CO_2_] for Takanari in the 0 N plot could not be identified in the present study. In an earlier study (Taylaran et al., 2009), showed that Takanari had significantly greater root length density than japonica cultivars, which might help to absorb N in the N-depleted soil. We speculate that E-[CO_2_] could enhance the ability of Takanari to collect N from the subsoil. However, there is a concern about the long-term sustainability of the soil N supplying capacity if Takanari-type cultivars are repeatedly grown, although there is limited evidence that long-term CO_2_ fumigation increases soil C and N accumulation (Guo et al., 2015). However, at least for the 3 years tested in the present study, the advantage of Takanari over Koshihikari was consistently evident.

There has been only limited evidence that E-[CO_2_] affects N_2_ fixation in the paddy (Hayashi et al., 2016); however, one previous study showed increased biological N_2_ fixation activity by E-[CO_2_] (Hoque et al., 2001). Free-living N_2_ fixation was not affected by E-[CO_2_] (Hayashi et al., 2014); however, there is a possibility that associative N_2_ fixation might be enhanced by E-[CO_2_]. Nitrification and denitrification processes are a large source of N loss in the paddy soil (Ishii et al., 2011; Bloom, 2015). There is growing evidence that genotypic difference in N use efficiency is related to the capacity to utilize or transport nitrate in the rhizosphere (Li et al., 2008; Hu et al., 2015) or to inhibit nitrification (Sun et al., 2016). Because we did not measure root morphology or rhizosphere chemistry of the two cultivars, we cannot discuss the relevance of the varietal difference in the N use efficiency in E-[CO_2_] observed in our results, but this is an important subject in the future. The long-term response of the soil-plant system to E-[CO_2_] is complex and uncertain (Reich and Hobbie, 2012; Reich et al., 2018). Better understandings of soil-plant dynamics that are affected by climatic and genetic factors are key to the effective development of long-term sustainable cultivars and management practices under climate change.

### Grain Quality Degradation and Quality Related Responses

Grain appearance quality of Koshihikari was substantially decreased by E-[CO_2_] by the increased occurrence of CG and quality degradation was particularly noted under the N limited condition (Table 5). The percentage of CG was higher in warmer years, suggesting that the quality degradation by E-[CO_2_] was similar to that frequently observed under heat during the grain filling period (Usui et al., 2016). Previous studies have shown that, under heat, the starch granules of endosperm cells become loosely packed and the air spaces between the granules create chalky appearances (Morita et al., 2016). It has been commonly observed that reduced N or protein content of the grain increases heat-induced CG (Morita et al., 2005; Wakamatsu et al., 2008), in particular, white-base and white-back type CG, which accounted for a majority (71 %) of the variation in the total CG observed in Koshihikari (Table S2). CG increased significantly with a decrease in PNC (Figure 5A). PNC was decreased not only by reduced N fertilization but also by E-[CO_2_], which has been commonly observed in many cereal crops (Myers et al., 2014). Consequently, the highest occurrence of CG was observed in the E-[CO_2_] and 0 N plot in the hottest summer in 2013 (Table 5). Elevated [CO_2_] increased CG partly via the reduced PNC; however, as shown in the relation between CG and PNC (Figure 5), the CG-PNC relation was significantly different between E-[CO_2_] and A-[CO_2_]. CG of E-[CO_2_] was larger than that of A-[CO_2_] at the same PNC, suggesting that other factors are involved in the increase in CG by E-[CO_2_].

Previous studies showed that E-[CO_2_] increased panicle temperature as a result of decreased leaf stomatal conductance and transpirational cooling by E-[CO_2_]; a typical increase in daytime panicle temperature was around 0.3–0.5°C (Yoshimoto et al., 2005). This might not be negligible for appearance quality when the temperature exceeds the threshold of approximately 26°C (Morita et al., 2016). However, it remains unclear how much panicle temperature and PNC account for the quality degradation under E-[CO_2_].

In contrast to Koshihikari, Takanari showed consistently low CG in all treatments and years (Table 5) although UDG was generally poor mainly due to malshaped grains. Recently, breeding efforts are in progress to improve appearance quality traits under high temperatures (heat tolerance) and Takanari is known for its resistance to heat for CG occurrence and is currently used as a donor to heat tolerance (Ishimaru et al., 2016). In our previous FACE study, we showed that Takanari retained a good appearance quality under E-[CO_2_] (Zhang et al., 2015a). Other studies revealed that Takanari had a consistently high stomatal conductance in both E- and A-[CO_2_] (Chen et al., 2014) and a lower canopy temperature by 0.4°C than Koshihikari (Ikawa et al., 2018). Therefore, the difference in canopy or panicle temperature between the two cultivars might partially account for the difference in CG.

The present study showed that CG of Takanari did not reveal any decrease in grain appearance quality or increase in CG under N limited conditions (Figure 5B). PNC of Takanari was decreased by E-[CO_2_] (Table 5); however, unlike Koshihikari, this decrease in PNC did not increase CG even during the hot summer. Takanari had a much lower % of white-base CG than Koshihikari (Table S2), which is known to be sensitive to N nutrition (Morita et al., 2005); this reduced the sensitivity of CG to PNC. Increases in heat-induced white-base CG can be decreased by additional N fertilization to retain higher PNC (Morita et al., 2016); however, increases in PNC can have negative effects on eating quality (Okadome, 2005). The low CG of Takanari even under low N conditions is thus a favorable trait to make both appearance and eating quality traits compatible.

Mechanistic understanding of the negative correlation between PNC and CG for standard japonica cultivars is still limited. A recent genetic and molecular study revealed that *sucrose synthase 3 (Sus3)* derived from an indica cultivar Habataki, a sister variety of Takanari, increases under high temperature, which confers heat tolerance for appearance quality (Takehara et al., 2018). The relevance of sucrose synthase and N availability is unknown; however, lower PNC might reduce this enzyme and possibly exacerbate appearance quality. It is unclear how Habataki *Sus3* performs under limited N condition; however, this could be effective even under low N availability. Several other quantitative trait loci for appearance quality have also been identified on chromosomes 3, 5, 8, and 12 (Takeuchi, 2016); however, the functions of these remain unknown. Future studies are required to determine genetic and molecular backgrounds for the CO_2_ × N × Variety interaction for appearance quality to effectively develop countermeasures against quality degradation under climate change.

## Conclusions

Our 3-year FACE experiment combined with three levels of N fertilizers revealed that the CO_2_ fertilization effect of a standard japonica cultivar, Koshihikari, diminished as N availability decreased, mainly because of sink limitation. However, sink limitation did not occur in Takanari even without N application, resulting in a significant yield enhancement of 18% by E-[CO_2_] in the 0N plot. Moreover, Takanari showed a consistently low CG in E-[CO_2_], whereas appearance quality of Koshihikari was severely damaged by E-[CO_2_], most notably in the 0N plot because of an increased occurrence of CG. These results indicated that Takanari could retain its yield advantage over Koshihikari even under limited N conditions and it could be a useful genetic resource for improving N use efficiency under E-[CO_2_].

## Author Contributions

TH, HS, TT, and KH identified the research question and designed the field experiments. TH, HS, TT, YU, CC, HI, HW, GZ, HfN, and KH conducted the field experiments and determined growth and yield traits. HsN operated the FACE facility and collected environmental data. HS conducted the chemical analysis. MM, TT, and KH conducted soil analysis. TH, HW, and CC conducted the data analysis. TH wrote the manuscript. All commented and approved the manuscript.

### Conflict of Interest Statement

The authors declare that the research was conducted in the absence of any commercial or financial relationships that could be construed as a potential conflict of interest.

## References

[B1] Bloom A. J. (2015). The increasing importance of distinguishing among plant nitrogen sources. Curr. Opin. Plant Biol. 25, 10–16. 10.1016/j.pbi.2015.03.002 25899331

[B2] Cassman K. G. Dobermann A. Walters D. T. Yang H. (2003). Meeting cereal demand while protecting natural resources and improving environmental quality. Annu. Rev. Environ. Resour. 28, 315–358. 10.1146/annurev.energy.28.040202.122858

[B3] Chen C. P. Sakai H. Tokida T. Usui Y. Nakamura H. Hasegawa T. (2014). Do the rich always become richer? Characterizing the leaf physiological response of the high-yielding rice cultivar takanari to free-air CO_2_ enrichment. Plant Cell Physiol. 55, 381–391. 10.1093/pcp/pcu009 24443497 PMC3913450

[B4] de Mendiburu F. (2017). Statistical Procedures for Agricultural Research. Available online at: https://cran.r-project.org/web/packages/agricolae/agricolae.pdf (accessed March 19, 2019).

[B5] Deryng D. Elliott J. Folberth C. Müller C. Pugh T. A. M. Boote K. J. (2016). Regional disparities in the beneficial effects of rising CO_2_ concentrations on crop water productivity. Nat. Clim. Chang. 6, 1–7. 10.1038/nclimate2995

[B6] Evans L. T. (1993). Crop Evolution, Adaptation and Yield. New York, NY: Cambridge University Press.

[B7] Feng Z. Rütting T. Pleijel H. Wallin G. Reich P. B. Kammann C. I.. (2015). Constraints to nitrogen acquisition of terrestrial plants under elevated CO_2_. Glob. Chang. Biol. 21, 3152–3168. 10.1111/gcb.12938 25846203

[B8] Guo J. Zhang M. Wang X. Zhang W. (2015). Elevated CO_2_ facilitates C and N accumulation in a rice paddy ecosystem. J. Environ. Sci. 29, 27–33. 10.1016/j.jes.2014.05.055 25766010

[B9] Hasegawa T. Li T. Yin X. Zhu Y. Boote K. Baker J.. (2017). Causes of variation among rice models in yield response to CO_2_ examined with free-air CO_2_ enrichment and growth chamber experiments. Sci. Rep. 7:14858. 10.1038/s41598-017-13582-y 29093514 PMC5666007

[B10] Hasegawa T. Sakai H. Tokida T. Nakamura H. Zhu C. Usui Y. (2013). Rice cultivar responses to elevated CO_2_ at two free-air CO_2_ enrichment (FACE) sites in Japan. Funct. Plant Biol. 40, 148–159. 10.1071/FP12357 32481095

[B11] Hasegawa T. Sakai H. Tokida T. Usui Y. Yoshimoto M. Fukuoka M. (2016). Rice free-air carbon dioxide enrichment studies to improve assessment of climate change effects on rice agriculture, in Improving Modeling Tools to Assess Climate Change Effects on Crop Response, eds Hatfield J. L. Fleisher D. (Madison, WI: American Society of Agronomy), 45–68. 10.2134/advagricsystmodel7.2014.0015

[B12] Hayashi K. Tokida T. Hasegawa T. (2016). FACEing up to future uncertainty : free-air CO_2_ enrichment experiments in Japanese rice paddy ecosystems, in The Challenges of Agro-Environmental Research in Monsoon Asia, eds Yagi K. Kuo C. G. (Tsukuba: National Institute for Agro-Environmental Sciences), 93–114.

[B13] Hayashi K. Tokida T. Matsushima M. Y. Ono K. Nakamura H. Hasegawa T. (2014). Free-air CO_2_ enrichment (FACE) net nitrogen fixation experiment at a paddy soil surface under submerged conditions. Nutr. Cycl. Agroecosystems 98, 57–69. 10.1007/s10705-013-9595-4

[B14] Hoque M. Inubushi K. Miura S. Kobayashi K. Kim H.-Y. Okada M. (2001). Biological dinitrogen fixation and soil microbial biomass carbon as influenced by free-air carbon dioxide enrichment (FACE) at three levels of nitrogen fertilization in a paddy field. Biol. Fertil. Soils 34, 453–459. 10.1007/s00374-001-0430-8

[B15] Hu B. Wang W. Ou S. Tang J. Li H. Che R.. (2015). Variation in NRT1.1B contributes to nitrate-use divergence between rice subspecies. Nat. Genet. 47, 834–838. 10.1038/ng.3337 26053497

[B16] Iizumi T. Furuya J. Shen Z. Kim W. Okada M. Fujimori S.. (2017). Responses of crop yield growth to global temperature and socioeconomic changes. Sci. Rep. 7:7800. 10.1038/s41598-017-08214-4 28798370 PMC5552729

[B17] Ikawa H. Chen C. P. Sikma M. Yoshimoto M. Sakai H. Tokida T.. (2018). Increasing canopy photosynthesis in rice can be achieved without a large increase in water use-A model based on free-air CO_2_ enrichment. Glob. Chang. Biol. 24, 1321–1341. 10.1111/gcb.13981 29136323

[B18] Imbe T. Akama Y. Nakane A. Hata T. Ise K. Ando I. (2004). Development of a multipurpose high-yielding rice variety Takanari. Bull. Natl Inst. Crop Sci. 5, 35–51.

[B19] Ishii S. Ikeda S. Minamisawa K. Senoo K. (2011). Nitrogen cycling in rice paddy environments: past achievements and future challenges. Microb. Environ. 26, 282–292. 10.1264/jsme2.me11293 22008507

[B20] Ishimaru T. Hirabayashi H. Sasaki K. Ye C. Kobayashi A. (2016). Breeding efforts to mitigate damage by heat stress to spikelet sterility and grain quality. Plant Prod. Sci. 19, 12–21. 10.1080/1343943X.2015.1128113

[B21] Kim H.-Y. Lieffering M. Kobayashi K. Okada M. Mitchell M. W. Gumpertz M. (2003). Effects of free-air CO_2_ enrichment and nitrogen supply on the yield of temperate paddy rice crops. Field Crop. Res. 83, 261–270. 10.1016/S0378-4290(03)00076-5

[B22] Kimball B. Kobayashi K. Bindi M. (2002). Responses of agricultural crops to free-air CO_2_ enrichment. Adv. Agron. 77, 293–368. 10.1016/S0065-2113(02)77017-X 12557686

[B23] Kimball B. A. (2016). Crop responses to elevated CO_2_ and interactions with H_2_O, N, and temperature. Curr. Opin. Plant Biol. 31, 36–43. 10.1016/j.pbi.2016.03.006 27043481

[B24] Kobayashi A. Hori K. Yamamoto T. Yano M. (2018). Koshihikari: a premium short-grain rice cultivar – its expansion and breeding in Japan. Rice 11:15. 10.1186/s12284-018-0207-4 29629486 PMC5890008

[B25] Kobayashi K. Okada M. Kim H. Y. Lieffering M. Miura S. Hasegawa T. (2006). Paddy Rice Responses to Free-Air [CO_2_] Enrichment, in Managed Ecosystems and CO_2_: Case Studies, Processes, and Perspectives, eds Nösberger J. Long S. P. Norby R. J. Stitt M. Hendrey G. R. Blum H. (Berlin; Heidelberg: Springer), 87–104.

[B26] Li Y. L. Fan X. R. Shen Q. R. (2008). The relationship between rhizosphere nitrification and nitrogen-use efficiency in rice plants. Plant Cell Environ. 31, 73–85. 10.1111/j.1365-3040.2007.01737.x 17944815

[B27] Morita S. Kusuda O. Yonemaru J. Fukushima A. Nakano H. (2005). Effects of topdressing on grain shape and grain damage under high temperature during ripening of rice, in Rice Is Life: Scientific Perspectives for the 21st Century, Proceedings of the World Rice Research Conference, Tsukuba, Japan, eds Toriyama K. Heong K. L. Hardy B. (Los Banos: International Rice Research Institute and Japan International Research Center for Agricultural Sciences), 560–562.

[B28] Morita S. Wada H. Matsue Y. (2016). Countermeasures for heat damage in rice grain quality under climate change. Plant Prod. Sci. 19, 1–11. 10.1080/1343943X.2015.1128114

[B29] Myers S. S. Zanobetti A. Kloog I. Huybers P. Leakey A. D. B. Bloom A. J.. (2014). Increasing CO_2_ threatens human nutrition. Nature 510, 139–142. 10.1038/nature13179 24805231 PMC4810679

[B30] Nakamura H. Tokida T. Yoshimoto M. Sakai H. Fukuoka M. Hasegawa T. (2012). Performance of the enlarged Rice-FACE system using pure CO_2_ installed in Tsukuba, Japan. J. Agric. Meteorol. 68, 15–23. 10.2480/agrmet.68.1.2

[B31] Nakano H. Makino A. Mae T. (1997). The effect of elevated partial pressures of CO_2_ on the relationship between photosynthetic capacity and N content in rice leaves. Plant Physiol. 115, 191–198. 12223799 10.1104/pp.115.1.191 PMC158474

[B32] Nakano H. Yoshinaga S. Takai T. Arai-Sanoh Y. Kondo K. Yamamoto T.. (2017). Quantitative trait loci for large sink capacity enhance rice grain yield under free-air CO_2_ enrichment conditions. Sci. Rep. 7:1827. 10.1038/s41598-017-01690-8 28500344 PMC5431863

[B33] Oikawa S. Ehara H. Koyama M. Hirose T. Hikosaka K. Chen C. P. (2017). Nitrogen resorption in senescing leaf blades of rice exposed to free-air CO_2_ enrichment (FACE) under different N fertilization levels. Plant Soil 418, 231–240. 10.1007/s11104-017-3280-3

[B34] Okadome H. (2005). Application of instrument-based multiple texture measurement of cooked milled-rice grains to rice quality evaluation. Japan Agric. Res. Q. 39, 261–268. 10.6090/jarq.39.261

[B35] Reich P. B. Hobbie S. E. (2012). Decade-long soil nitrogen constraint on the CO_2_ fertilization of plant biomass. Nat. Clim. Chang. 2, 1–5. 10.1038/nclimate1694

[B36] Reich P. B. Hobbie S. E. Lee T. D. Pastore M. A. (2018). Unexpected reversal of C_3_ versus C4 grass response to elevated CO_2_ during a 20-year field experiment. Science 360, 317–320. 10.1126/SCIENCE.AAS9313 29674593

[B37] Rosenzweig C. Elliott J. Deryng D. Ruane A. C. Müller C. Arneth A.. (2014). Assessing agricultural risks of climate change in the 21st century in a global gridded crop model intercomparison. Proc. Natl. Acad. Sci. U.S.A. 111, 3268–3273. 10.1073/pnas.1222463110 24344314 PMC3948251

[B38] Sakai H. Hasegawa T. Kobayashi K. (2006). Enhancement of rice canopy carbon gain by elevated CO_2_ is sensitive to growth stage and leaf nitrogen concentration. New Phytol. 170, 321–332. 10.1111/j.1469-8137.2006.01688.x 16608457

[B39] Schleussner C.-F. Deryng D. Müller C. Elliott J. Saeed F. Folberth C. (2018). Crop productivity changes in 1.5°C and 2°C worlds under climate sensitivity uncertainty. Environ. Res. Lett. 13:064007 10.1088/1748-9326/aab63b

[B40] Stitt M. Krapp A. (1999). The interaction between elevated carbon dioxide and nitrogen nutrition: the physiological and molecular background. Plant Cell Environ. 22, 583–621. 10.1046/j.1365-3040.1999.00386.x

[B41] Sun L. Lu Y. Yu F. Kronzucker H. J. Shi W. (2016). Biological nitrification inhibition by rice root exudates and its relationship with nitrogen-use efficiency. New Phytol. 212, 646–656. 10.1111/nph.14057 27292630

[B42] Takai T. Ikka T. Kondo K. Nonoue Y. Ono N. Arai-Sanoh Y.. (2014). Genetic mechanisms underlying yield potential in the rice high-yielding cultivar Takanari, based on reciprocal chromosome segment substitution lines. BMC Plant Biol. 14:295. 10.1186/s12870-014-0295-2 25404368 PMC4243286

[B43] Takai T. Nakano H. Yoshinaga S. Kondo M. (2018). Identification of a novel QTL for the number of spikelets per panicle using a cross between *indica*- and *japonica*-type high-yielding rice cultivars in Japan. Plant Breed. 137, 109–117. 10.1111/pbr.12575

[B44] Takehara K. Murata K. Yamaguchi T. Yamaguchi K. Chaya G. Kido S. (2018). Thermo-responsive allele of *sucrose synthase* 3 (*Sus3*) provides high-temperature tolerance during the ripening stage in rice (*Oryza sativa* L.). Breed. Sci. 342, 336–342. 10.1270/jsbbs.18007 PMC6081304 30100800

[B45] Takeuchi Y. (2016). Development of isogenic lines of Koshihikari with heat tolerant QTLs. -QTLs derived from Takanari and Ma Li Xian, in Kenkyuseika554 Development of Mitigation and Adaptation Technologies to Climate Change in the Sectors of Agriculture, Forestry, and Fisheries - Development of Gramineous Crop Cultivars Adapted Global Warming - (Tokyo: Ministry of Agriculture Forestry and Fisheries), 40–42.

[B46] Taylaran R. Ozawa S. Miyamoto N. (2009). Performance of a high-yielding modern rice cultivar Takanari and several old and new cultivars grown with and without chemical fertilizer in a submerged paddy field. Plant Prod. Sci. 12, 365–380. 10.1626/pps.12.365

[B47] Toriyama K. (2002). Estimation of fertilizer nitrogen requirement for average rice yield in Japanese paddy fields. Soil Sci. Plant Nutr. 48, 293–300. 10.1080/00380768.2002.10409204

[B48] Usui Y. Sakai H. Tokida T. Nakamura H. Nakagawa H. Hasegawa T. (2014). Heat-tolerant rice cultivars retain grain appearance quality under free-air CO_2_ enrichment. Rice 7:6. 10.1186/s12284-014-0006-5 24920972 PMC4052674

[B49] Usui Y. Sakai H. Tokida T. Nakamura H. Nakagawa H. Hasegawa T. (2016). Rice grain yield and quality responses to free-air CO_2_ enrichment combined with soil and water warming. Glob. Chang. Biol. 22, 1256–1270. 10.1111/gcb.13128 26463894

[B50] Wada G. (1969). The effect of nitrogen nutrition on the yield-determining process of rice plant. Bull. Natl. Inst. Agric. Sci. Jpn. Ser. A 16, 1–271.

[B51] Wakamatsu K. Sasaki O. Uezono I. Tanaka A. (2008). Effect of the amount of nitrogen application on occurrence of white-back kernels during ripening of rice under high-temperature conditions. Japanese J. Crop Sci. 77, 424–433. 10.1626/jcs.77.424

[B52] Wang F. Peng S. B. (2017). Yield potential and nitrogen use efficiency of China's super rice. J. Integr. Agric. 16, 1000–1008. 10.1016/S2095-3119(16)61561-7

[B53] Yang L. Huang J. Yang H. Zhu J. Liu H. Dong G. (2006). The impact of free-air CO_2_ enrichment (FACE) and N supply on yield formation of rice crops with large panicle. F. Crop. Res. 98, 141–150. 10.1016/j.fcr.2005.12.014

[B54] Yang L. Wang Y. Dong G. Gu H. Huang J. Zhu J. (2007). The impact of free-air CO_2_ enrichment (FACE) and nitrogen supply on grain quality of rice. Field Crop. Res. 102, 128–140. 10.1016/j.fcr.2007.03.006

[B55] Yoshimoto M. Oue H. Takahashi N. (2005). The effects of FACE (Free-Air CO_2_ Enrichment) on temperatures and transpiration of rice panicles at flowering stage. J. Agric. Meteorol. 60, 597–600. 10.2480/agrmet.597

[B56] Zhang G. Sakai H. Tokida T. Usui Y. Zhu C. Nakamura H. (2013). The effects of free-air CO_2_ enrichment (FACE) on carbon and nitrogen accumulation in grains of rice (*Oryza sativa* L.). J. Exp. Bot. 64, 3179–3188. 10.1093/jxb/ert154 23918962 PMC3733142

[B57] Zhang G. Sakai H. Usui Y. Tokida T. Nakamura H. Zhu C. (2015a). Grain growth of different rice cultivars under elevated CO_2_ concentrations affects yield and quality. Field Crop. Res. 179, 72–80. 10.1016/j.fcr.2015.04.006

[B58] Zhang X. Davidson E. A. Mauzerall D. L. Searchinger T. D. Dumas P. Shen Y. (2015b). Managing nitrogen for sustainable development. Nature 528, 51–59. 10.1038/nature15743 26595273

[B59] Zhao C. Liu B. Piao S. Wang X. Lobell D. B. Huang Y.. (2017). Temperature increase reduces global yields of major crops in four independent estimates. Proc. Natl. Acad. Sci. U.S.A. 114, 9326–9331. 10.1073/PNAS.1701762114 28811375 PMC5584412

[B60] Zhu C. Xu X. Wang D. Zhu J. Liu G. (2015). An indica rice genotype showed a similar yield enhancement to that of hybrid rice under free air carbon dioxide enrichment. Sci. Rep. 5:15312 10.1038/srep12719 26228872 PMC4521144

