# Supplementary material for: A High-Yielding Rice Cultivar “Takanari” Shows No N Constraints on CO2 Fertilization
Source: Front Plant Sci. 2019 Apr 5;10:361. doi: 10.3389/fpls.2019.00361 (PMC6460941; doi:10.3389/fpls.2019.00361)
Supplement: Supplementary file 1 [file Data_Sheet_1.docx]

Supplementary Material

| Table S1. The effects of [CO_2_] and N on spikelet number per biomass and aboveground crop N of Koshihikari and Takanari measured at maturity, along with the standard deviation (n=4) | | | | | | | | | | | | | | | |
| --- | --- | --- | --- | --- | --- | --- | --- | --- | --- | --- | --- | --- | --- | --- | --- |
| Cultivar | Year | N (g m^-2^) | Spikelet No/Biomass (g^-1^) | | | | | |  | Aboveground Crop  N (g m^-2^) | | | | | |
|  |  |  | A-[CO_2_] | | | E-[CO_2_] | | |  | A-[CO_2_] | | | E-[CO_2_] | | |
| Koshihikari | 2012 | 0 | 21.4 | ± | 0.3 | 21.1 | ± | 0.6 |  | 6.2 | ± | 0.5 | 5.9 | ± | 0.7 |
|  |  | 8 | 23.5 | ± | 0.7 | 23.8 | ± | 1.7 |  | 11.2 | ± | 0.9 | 10.5 | ± | 0.7 |
|  |  | 12 | 26.5 | ± | 0.6 | 23.9 | ± | 0.8 |  | 14.2 | ± | 1.8 | 13.2 | ± | 1.7 |
|  | 2013 | 0 | 22.1 | ± | 0.3 | 21.0 | ± | 0.6 |  | 6.4 | ± | 0.7 | 6.3 | ± | 0.7 |
|  |  | 8 | 22.1 | ± | 0.8 | 22.7 | ± | 0.8 |  | 11.0 | ± | 0.7 | 11.2 | ± | 0.7 |
|  |  | 12 | 23.3 | ± | 0.3 | 22.7 | ± | 0.5 |  | ND^1)^ |  |  | ND |  |  |
|  | 2014 | 0 | 23.2 | ± | 1.2 | 22.1 | ± | 0.5 |  | 6.8 | ± | 0.4 | 6.6 | ± | 0.8 |
|  |  | 8 | 24.7 | ± | 0.8 | 23.6 | ± | 1.3 |  | 10.1 | ± | 0.4 | 9.6 | ± | 1.1 |
|  |  | 12 | 27.0 | ± | 0.7 | 25.9 | ± | 1.8 |  | 13.2 | ± | 0.4 | 12.7 | ± | 1.9 |
|  | mean | 0 | 22.3 |  |  | 21.4 |  |  |  | 6.5 |  |  | 6.3 |  |  |
|  |  | 8 | 23.4 |  |  | 23.4 |  |  |  | 10.8 |  |  | 10.4 |  |  |
|  |  | 12 | 25.6 |  |  | 24.2 |  |  |  | 13.7 |  |  | 13.0 |  |  |
| Takanari | 2012 | 0 | 30.1 | ± | 1.4 | 29.1 | ± | 1.4 |  | 5.8 |  | 1.0 | 6.4 | ± | 0.5 |
|  |  | 8 | 29.5 | ± | 2.0 | 29.6 | ± | 0.5 |  | 10.7 | ± | 1.1 | 10.8 | ± | 0.6 |
|  |  | 12 | 28.2 | ± | 1.2 | 27.1 | ± | 1.4 |  | ND |  |  | ND |  |  |
|  | 2013 | 0 | 28.4 | ± | 0.5 | 28.4 | ± | 0.9 |  | 7.7 | ± | 1.2 | 7.7 | ± | 0.7 |
|  |  | 8 | 27.5 | ± | 0.6 | 27.0 | ± | 0.9 |  | 11.9 | ± | 0.7 | 12.3 | ± | 0.8 |
|  |  | 12 | 27.9 | ± | 0.9 | 27.9 | ± | 0.5 |  | ND |  |  | ND |  |  |
|  | 2014 | 0 | 29.4 | ± | 1.3 | 29.4 | ± | 0.9 |  | 6.9 | ± | 0.6 | 7.3 | ± | 1.1 |
|  |  | 8 | 29.9 | ± | 0.3 | 30.1 | ± | 0.4 |  | 10.4 | ± | 1.3 | 12.2 | ± | 1.2 |
|  |  | 12 | 29.7 | ± | 0.8 | 29.6 | ± | 0.6 |  | 12.6 | ± | 0.7 | 15.0 | ± | 2.5 |
|  | mean | 0 | 29.3 |  |  | 28.9 |  |  |  | 6.8 |  |  | 7.1 |  |  |
|  |  | 8 | 28.9 |  |  | 28.9 |  |  |  | 11.0 |  |  | 11.8 |  |  |
|  |  | 12 | 28.6 |  |  | 28.2 |  |  |  | 12.6 |  |  | 15.0 |  |  |
| ANOVA ^2)^ |  |  | 3 years with 3 N levels | | | | | | 3 years with 2 N levels (0N and 8N) | | | | 2014 with 3 N levels | | |
| Year |  |  | *** | | | | | | * | | | |  | | |
| CO_2_ |  |  | * | | | | | | ns | | | | ns | | |
| CO_2_×Year |  |  | ns | | | | | | ns | | | |  | | |
| N |  |  | *** | | | | | | *** | | | | *** | | |
| N× CO_2_ |  |  | 0.053 | | | | | | ns | | | | ns | | |
| N×Year |  |  | ** | | | | | | 0.078 | | | |  | | |
| N×Year × CO_2_ | |  | ns | | | | | | ns | | | |  | | |
| Variety (V) |  |  | *** | | | | | | *** | | | | ** | | |
| V × Year |  |  | ns | | | | | | ** | | | |  | | |
| V × CO_2_ |  |  | ns | | | | | | ** | | | | ** | | |
| V × N |  |  | *** | | | | | | ns | | | | ns | | |
| V × Year × CO_2_ | |  | ns | | | | | | ns | | | |  | | |
| V × Year × N | |  | *** | | | | | | ns | | | |  | | |
| V × CO_2_ × N |  |  | ns | | | | | | ns | | | | ns | | |
| V × CO_2_ × N ×Year |  |  | ns | | | | | | ns | | | |  | | |
| 1) ND, No data (not measured). | | | |  |  |  |  |  |  |  |  |  |  |  |  |
| 2) ANOVA was performed using all years and treatment combinations for spikelet number per biomass. For aboveground crop N, two ANOVA results are presented to avoid missing cells: one with 3 years with 0 and 8N and the other with 3N in 2014. Both results agreed in the significance of the treatment effects without interactions with years. | | | | | | | | | | | | | | | |

| Table S2.The effects of [CO_2_] and N on different types of chalky grains of Koshihikari and Takanari along with the standard deviation (n=4) | | | | | | | | | | | | | | | | | | | | | | |
| --- | --- | --- | --- | --- | --- | --- | --- | --- | --- | --- | --- | --- | --- | --- | --- | --- | --- | --- | --- | --- | --- | --- |
| Cultivar | Year | N (g m^-2^) | White belly(%) | | | | | |  | White Base (%) | | | | | |  | Milky white (%) | | | | | |
|  |  |  | A-[CO_2_] | | | E-[CO_2_] | | |  | A-[CO_2_] | | | E-[CO_2_] | | |  | A-[CO_2_] | | | E-[CO_2_] | | |
| Koshihikari | 2012 | 0 | 1.4 | ± | 0.2 | 2.2 | ± | 0.8 |  | 10.4 | ± | 1.6 | 18.5 | ± | 2.3 |  | 2.3 | ± | 0.3 | 3.5 | ± | 1.2 |
|  |  | 8 | 1.5 | ± | 1.0 | 1.9 | ± | 0.4 |  | 5.1 | ± | 2.1 | 9.8 | ± | 2.2 |  | 2.0 | ± | 0.5 | 3.0 | ± | 1.7 |
|  |  | 12 | 2.4 | ± | 0.4 | 2.0 | ± | 0.5 |  | 2.6 | ± | 1.1 | 8.2 | ± | 1.9 |  | 7.8 | ± | 1.4 | 4.9 | ± | 0.4 |
|  | 2013 | 0 | 7.9 | ± | 1.4 | 9.6 | ± | 0.7 |  | 32.3 | ± | 1.7 | 31.3 | ± | 2.2 |  | 11.4 | ± | 2.6 | 19.0 | ± | 1.9 |
|  |  | 8 | 3.4 | ± | 0.5 | 5.4 | ± | 1.1 |  | 22.4 | ± | 1.6 | 32.5 | ± | 6.7 |  | 7.5 | ± | 1.3 | 12.2 | ± | 1.9 |
|  |  | 12 | 4.3 | ± | 1.1 | 6.1 | ± | 0.7 |  | 18.0 | ± | 5.8 | 34.3 | ± | 1.9 |  | 11.5 | ± | 0.9 | 15.1 | ± | 2.0 |
|  | 2014 | 0 | 3.5 | ± | 1.1 | 5.7 | ± | 0.6 |  | 19.3 | ± | 4.1 | 36.5 | ± | 3.6 |  | 5.6 | ± | 0.9 | 10.1 | ± | 0.5 |
|  |  | 8 | 2.9 | ± | 0.6 | 4.7 | ± | 0.4 |  | 9.2 | ± | 2.5 | 23.1 | ± | 4.0 |  | 9.3 | ± | 1.4 | 10.1 | ± | 1.6 |
|  |  | 12 | 2.4 | ± | 0.8 | 5.0 | ± | 0.6 |  | 4.2 | ± | 1.2 | 17.6 | ± | 5.2 |  | 11.0 | ± | 2.0 | 12.9 | ± | 2.8 |
|  | mean | 0 | 4.2 |  |  | 5.8 |  |  |  | 20.7 |  |  | 28.8 |  |  |  | 6.4 |  |  | 10.9 |  |  |
|  |  | 8 | 2.6 |  |  | 4.0 |  |  |  | 12.2 |  |  | 21.8 |  |  |  | 6.3 |  |  | 8.4 |  |  |
|  |  | 12 | 3.0 |  |  | 4.4 |  |  |  | 8.3 |  |  | 20.0 |  |  |  | 10.1 |  |  | 10.9 |  |  |
| Takanari | 2012 | 0 | 1.2 | ± | 0.6 | 1.0 | ± | 0.4 |  | 2.3 |  | 0.8 | 2.4 | ± | 1.0 |  | 1.1 | ± | 0.3 | 1.4 | ± | 0.7 |
|  |  | 8 | 1.8 | ± | 0.3 | 2.4 | ± | 1.0 |  | 2.4 | ± | 0.9 | 2.8 | ± | 0.9 |  | 3.6 | ± | 1.0 | 3.1 | ± | 1.4 |
|  |  | 12 | ND^1)^ | | | ND | | |  | ND | | | ND | | |  | ND | | | ND | | |
|  | 2013 | 0 | 4.0 | ± | 1.5 | 5.7 | ± | 1.7 |  | 4.2 | ± | 1.7 | 4.1 | ± | 0.8 |  | 4.9 | ± | 0.9 | 5.7 | ± | 1.0 |
|  |  | 8 | 6.6 | ± | 1.8 | 7.5 | ± | 1.0 |  | 3.4 | ± | 1.2 | 5.8 | ± | 2.5 |  | 6.4 | ± | 1.7 | 7.7 | ± | 0.8 |
|  |  | 12 | 4.1 | ± | 1.6 | 8.1 | ± | 0.9 |  | 2.0 |  | 0.3 | 3.5 |  | 0.7 |  | 6.3 |  | 1.4 | 8.4 |  | 1.6 |
|  | 2014 | 0 | 2.0 | ± | 0.8 | 3.7 | ± | 0.9 |  | 2.7 | ± | 1.0 | 5.8 | ± | 2.4 |  | 4.1 | ± | 1.1 | 7.9 | ± | 3.0 |
|  |  | 8 | 4.0 | ± | 1.4 | 5.1 | ± | 1.1 |  | 3.3 | ± | 0.7 | 3.9 | ± | 1.7 |  | 9.7 | ± | 0.9 | 10.7 | ± | 1.5 |
|  |  | 12 | 3.5 | ± | 0.9 | 5.5 | ± | 1.2 |  | 2.6 | ± | 0.4 | 4.9 | ± | 0.5 |  | 10.6 | ± | 1.4 | 13.5 | ± | 0.5 |
|  | mean | 0 | 2.4 |  |  | 3.5 |  |  |  | 3.1 |  |  | 4.1 |  |  |  | 3.4 |  |  | 5.0 |  |  |
|  |  | 8 | 4.1 |  |  | 5.0 |  |  |  | 3.0 |  |  | 4.1 |  |  |  | 6.6 |  |  | 7.2 |  |  |
|  |  | 12 | 3.8 |  |  | 6.8 |  |  |  | 2.3 |  |  | 4.2 |  |  |  | 8.4 |  |  | 10.9 |  |  |
| 1) ND, No data (not measured). | |  |  |  |  |  |  |  |  |  |  |  |  |  |  |  |  |  |  |  |  |  |
